# Validation of preferred salt concentration in soup based on a randomized blinded experiment in multiple regions in Japan—influence of umami (l-glutamate) on saltiness and palatability of low-salt solutions

**DOI:** 10.1038/s41440-020-0397-1

**Published:** 2020-01-29

**Authors:** Hitomi Hayabuchi, Rieko Morita, Masanori Ohta, Akiko Nanri, Hideki Matsumoto, Shoji Fujitani, Shintaro Yoshida, Sadayoshi Ito, Atsushi Sakima, Hiroyuki Takase, Miho Kusaka, Takuya Tsuchihashi

**Affiliations:** 1 Department of Food Science and Nutrition, Faculty of Human Life and Environment, Nara Women’s University, Nara, Japan; 2 Graduate School of Health and Environmental Sciences, Fukuoka Women’s University, Fukuoka, Japan; 3 Research Institute for Bioscience Products & Fine Chemicals, Ajinomoto Co., Inc, Kawasaki, Japan; 4 Division of Nephrology, Endocrinology and Hypertension, Tohoku University Graduate Medicine, Sendai, Japan; 5 Department of Medicine, Katta General Hospital, Miyagi, Japan; 6 Health Administration Center, University of the Ryukyus, Okinawa, Japan; 7 Enshu Hospital, Hamamatsu, Japan; 8 Kusaka Clinic, Kure, Japan; 9 Steel Memorial Yawata Hospital, Kitakyushu, Japan

**Keywords:** Umami, Glutamate, Palatability, Sodium reduction, Randomized blind experiment

## Abstract

Sodium reduction is an important public health goal. Individual and population approaches are necessary for reducing the sodium content of processed foods and meals. The aim of the present study is to affirm the effect of monosodium l-glutamate (MSG), an umami substance, on the saltiness or palatability of low-salt solutions and to explore the preferred salt concentration in soup. Five hundred and eighty-four healthy participants from nineteen regions in Japan tasted 0.3, 0.6, and 0.9% NaCl solutions with or without 0.3% MSG. Evaluations of saltiness and palatability for each solution were conducted using a visual analog scale in a double-blinded randomized manner. Saltiness gradually increased depending on the concentration of NaCl. The saltiness of the 0.3% NaCl solution with MSG was rated significantly higher than that without MSG. The palatability ratings were higher for the solutions with MSG than for those without MSG for all NaCl concentrations. In particular, the palatability rating of the 0.3% NaCl solution with MSG was twice as high as that without MSG and was significantly higher than that of the other five test solutions. Furthermore, these results were observed to be approximately the same, irrespective of sex, age, region, etc. Salt reduction is believed to result in a loss of palatability. However, our results suggest that umami can compensate for the loss of palatability caused by salt reduction and that the addition of an appropriate amount of an umami substance can facilitate salt reduction from 0.9 to 0.3% without sacrificing palatability.

## Introduction

Reducing salt intake is essential for preventing the onset and aggravation of hypertension [1–4] and improving healthy longevity [5–7]. Higher sodium intake is reported to be associated with higher blood pressure [8–12]. According to the Global Burden of Disease project [13], elevated blood pressure (systolic > 115 mmHg) continues to be the largest single contributor to disease burden and mortality worldwide, resulting in 9.4 million deaths each year. Moreover, the global prevalence of hypertension is predicted to increase from 26.4% in 2000 to 29.2% by 2025, meaning that 1.56 billion people may be diagnosed with hypertension [14]. Another report also suggests that the number of people suffering from high blood pressure and the prevalence of high blood pressure are expected to continue increasing worldwide over the next decade [15]. Therefore, strategies to prevent hypertension are urgently needed.

In 2019, the Japanese Society of Hypertension published Guidelines for the Management of Hypertension [16]. The importance of lifestyle modifications for the management and prevention of hypertension is discussed in this guideline, and it is proposed that a salt reduction goal of <6 g per day should be set. However, according to the National Health and Nutrition Survey Japan in 2017 [17], the average daily salt intake was 10.8 g/day and 9.1 g/day in adult men and women, respectively. These intake levels are extremely far from the salt reduction goal proposed above and do not even approach the provisional dietary goal (daily salt intake: <8.0 g/day in men and <7.0 g/day in women) declared in the Dietary Reference Intakes for Japanese 2015, which was published with the aim of preventing noncommunicable diseases, including stroke, and cardiovascular diseases [18]. The daily salt intake recommendation of the World Health Organization (WHO) for adults to reduce blood pressure and the risk of cardiovascular disease, stroke and coronary heart disease is <5 g/day [19]. In addition, global targets established by the United Nations and the WHO for the prevention of chronic diseases include a 30% relative reduction in population-level salt intake by 2025 [20]. Many countries have initiated programs targeting salt reduction [21].

The positive correlation between sodium intake and systolic blood pressure has been shown in randomized controlled trials [22] and a meta-analysis [23]. To prevent and control prehypertension and hypertension, major reductions in the salt content of the food supply, including processed foods and restaurant meals, are needed.

The potential for a reduction in the NaCl content in foods to diminish their palatability is well known [24, 25]. Although salt reduction programs have been developed in many countries, few people pay attention to these programs and reduce their salt intake [26]. In addition, Kawamura et al. described the difficulty in convincing subjects to continue consuming a low-salt diet even after their participation in dietary programs and nutritional education for salt intake reduction [27]. Kubota et al. also indicated the possibility that impairment of salty taste recognition was associated with masked hypertension in women showing normal blood pressure during clinical examination [28]. Consequently, it is essential to find an appropriate compound to enhance palatability and determine its optimum levels to compensate for the palatability loss caused by salt reduction [1]. The addition of an umami substance (monosodium l-glutamate (MSG) or calcium diglutamate (CDG)) has made it possible to lower the NaCl concentration without sacrificing the pleasantness, saltiness, or taste intensity of soups [29–36]. In Japan, miso soup is an ordinary food item in the daily diet, and Takimoto et al. suggested that miso, or fermented soybean paste, is a prominent contributor to reducing daily salt intake [37]. Soup is well known to be a common food consumed not only in Japan but also throughout the world. It is therefore essential to control the NaCl concentration in soups to reduce daily salt consumption [30, 32, 35, 36]. In previous studies on the effects of MSG and NaCl concentration on palatability, broth made from dried bonito [29], chicken broth [30, 32, 34], vegetable soup [31, 33] or spicy chicken soup [35] were used as test objects, but limiting factors such as age and number of panelists were found. In this study, we investigated the influence of an umami substance on the saltiness and palatability of a low-salt solution prepared with distilled water as a medium and clarified the most preferred NaCl and MSG concentrations in a randomized blind experiment with participants from multiple regions in Japan.

## Methods

### Study procedure

This multicenter study was conducted from 2017 to 2018 in Japan, and participants comprised students from the nutrition departments of eight universities and attendees of 11 health seminars. Sensory evaluations were performed at eight universities located in Aomori, Chiba, Kanagawa, Shizuoka, Hiroshima, Fukuoka, and Kagoshima Prefectures in June 2017 and in Nara Prefecture in April 2018. The same general middle-aged and elderly subjects performed sensory evaluation tests at 11 health seminars held in Miyagi, Saitama, Tokyo, Shizuoka, Nara, Hiroshima, Fukuoka (Kitakyushu, Munakata, and Fukuoka City), Nagasaki, and Okinawa Prefectures from July to November 2018.

This study was conducted according to the guidelines presented in the Declaration of Helsinki, and all the subjects signed the informed consent form prior to participating in the study. All sensory evaluations were performed under the same protocol, which was approved by the Research Ethics Committee of Fukuoka Women’s University (Approval No: 2016-31) for students and the Research Ethics Review Committee at Nara Women’s University (Approval No: 18-02) for general adults. These sensory evaluations were registered with the University Hospital Medical Information Network Clinical Trials Registry System (UMIN000035280 and UMIN000035289, respectively).

To unify the conditions of the evaluation tests, we provided all items required for the sensory evaluation test, which included six concentrated solutions, distilled water for dilution, six reservoirs, six muddlers, small cups for dispensing six samples, clean water for mouth rinse, paper cups, consent forms, questionnaires, evaluation sheets, a thermometer, a survey implementation manual, and a sheet for recording.

On the day of the survey or the day before, staff performing the sensory evaluations diluted the concentrated solutions by adding distilled water and prepared test solutions with six cups labeled from A to F, according to the instructions in the procedure manual. They also recorded variables, such as weather, room temperature, water temperature, and the number of participants, in detail. All participants first evaluated the six test solutions, answered questionnaires and then performed a second sensory evaluation. During the first taste evaluation, six solutions with different concentrations were tasted in order from A to F in a double-blind manner. Participants were asked to rinse their mouth with clean water before tasting and spit out the solutions after tasting. The salinity and palatability intensity of each solution were assessed using a visual analog scale (VAS). Then, participants were asked to fill out the questionnaire about characteristics, smoking, etc. Finally, participants tasted the six solutions again from F to A and evaluated their saltiness and palatability.

### Participants

**Fig. 1:**
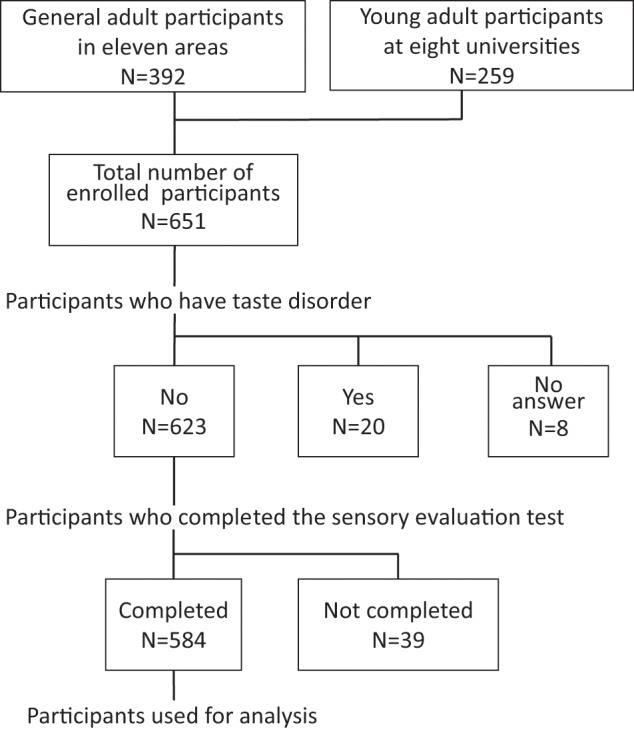
Flow chart of the analyzed participants

The participants in this study consisted of students from eight universities (*n* = 259) and adult attendees of 11 health seminars (*n* = 392). Of the 651 participants who performed evaluation tests, we excluded the participants who indicated that they had a taste disorder (*n* = 20), those who did not answer the question on taste disorder in the questionnaire (*n* = 8) and those who did not complete the sensory evaluation test (*n* = 39). Finally, a total of 584 men and women were included in the analysis (Fig. 1).

### Test samples

The test samples were six aqueous solutions containing three different concentrations of NaCl (0.3, 0.6, and 0.9%) with or without MSG (0.3%). NaCl was provided by the Salt Industry Center of Japan (>99%, Tokyo, Japan). MSG was provided by Ajinomoto Co., Inc. (>99%, Tokyo, Japan). Distilled water was provided by Kyoei Pharmaceutical Co., Inc. (Chiba, Japan). The six solutions were assigned randomly from A to F, and a 10 ml aliquot of each solution was dispensed.

Previously, we examined the saltiness, umami, and palatability of 48 aqueous solutions containing eight different concentrations of NaCl (0.2, 0.3, 0.4, 0.5, 0.6, 0.7, 0.8, and 0.9%) and six different concentrations of MSG (0.1, 0.2, 0.3, 0.4, 0.5, and 0.6%) by sensory evaluation among female students and teachers (unpublished data). As a result, the 0.6% NaCl and 0.3% MSG aqueous solutions were confirmed to have the highest palatability. In addition, the salt concentration of soup consumed by Japanese individuals is generally 0.9%. Based on the results of the previous examination, these aqueous solutions were prepared for the present study.

### Sensory evaluations

Saltiness and palatability were assessed by a VAS. A VAS is usually used as an appropriate method for evaluating the sensation induced by tastes and is confirmed to have reproducibility and validity in assessment [38]. In this study, we conducted sensory evaluation with a modification of the method performed by Carter et al. [34]. The scale was a 100-mm line labeled at the left end with the most negative or lowest intensity of perception (not at all salty or extremely unpalatable) and at the right end with opposing terms (extremely salty or palatable). After receiving an explanation of the VAS assessment method with examples, participants used a pen or pencil to position a point along the 100-mm line that described the taste intensity or palatability of each sample. These evaluations were carried out twice, changing the order of the samples after 15 min or more. The average of the two evaluations was adopted as the intensity of the tastes and palatability of the solutions and was used for analysis.

### Statistical analysis

Data are presented as the means and standard errors (SEs) unless stated otherwise. The first and second taste evaluations of the saltiness and palatability of the six aqueous solutions were averaged. Saltiness and palatability were analyzed using repeated measures analysis of variance. Further pairwise comparisons were made using the Tukey honestly significance difference procedure. Statistical significance was defined as *p* < 0.05 in all comparisons. Statistical analysis was conducted using JMP statistical software (JMP14.2, SAS Institute Inc., Cary, NC, USA).

## Results

### Implementation status and participants

Of the 19 facilities, 14 facilities conducted sensory evaluations at ~11:00 a.m., and the rest conducted sensory evaluations at ~3:00 p.m. The mean room temperature was 25.5 °C (±1.26 standard deviation (SD)), and the mean water temperature was 23.7 °C (±1.92 SD).

The characteristics of the study participants are shown in Table 1. The majority of participants were female (88.1%) nonsmokers without experience (92.3%) who had not eaten for more than 2 h after their last meal (77.6%). A total of 44.0% of participants were under 30 years old, and 34.1% took medication (63.2% over 60 years old). The percentages of participants by region were 11.6% in Tohoku, 24.7% in Kanto–Tokai, 19.7% in Kinki–Chugoku, 32.0% in North Kyushu, and 12.0% in South Kyushu.

**Table 1:** Characteristics of the study participants

Characteristics	Male *N* (%)	Female *N* (%)	All *N* (%)
All	69 (100)	515 (100)	584 (100)
Age group^a^			
19–29	6 (8.7)	251 (48.9)	257 (44.0)
30–59	17 (24.6)	55 (10.7)	72 (12.3)
60–69	21 (30.4)	79 (15.4)	100 (17.1)
>70	25 (36.2)	128 (25.0)	153 (26.2)
Smoking^a^			
Smoker	7 (10.1)	7 (1.4)	14 (2.4)
Past smoker	20 (29.0)	8 (1.6)	28 (4.8)
Never smoker	40 (58.0)	499 (96.9)	539 (92.3)
Fasting time^a^			
≤2 h	24 (34.8)	105 (20.4)	129 (22.1)
>2 h	45 (65.2)	408 (79.2)	453 (77.6)
Medicine^a^			
With medication	36 (52.2)	163 (31.7)	199 (34.1)
No medication	31 (44.9)	351 (68.2)	382 (65.4)
Area^b^			
Tohoku	9 (13.0)	59 (11.5)	68 (11.6)
Kanto–Tokai	14 (20.3)	130 (25.2)	144 (24.7)
Kinki–Chugoku	15 (21.7)	100 (19.4)	115 (19.7)
North Kyushu	30 (43.5)	157 (30.5)	187 (32.0)
South Kyushu	1 (1.4)	69 (13.4)	70 (12.0)

^a^Data exclude the participants who did not answer the questionnaire

^b^Tohoku includes Aomori and Miyagi; Kanto–Tokai includes Chiba, Saitama, Kanagawa, Tokyo and two areas in Shizuoka; Kinki–Chugoku includes two areas each in Nara and Hiroshima; North Kyushu includes Kitakyushu, Munakata, two areas in Fukuoka and Nagasaki; and South Kyushu includes Kagoshima and Okinawa

### Saltiness

**Fig. 2:**
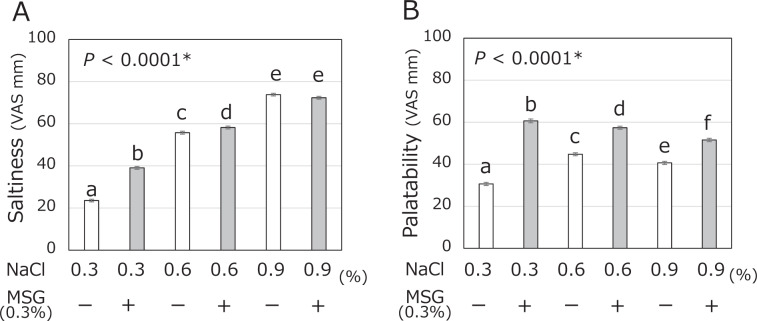
Mean and SE of saltiness (**A**) and palatability (**B**) VAS ratings obtained from the sensory evaluation. The VAS ratings of saltiness and palatability of six samples containing three different concentrations of NaCl (0.3, 0.6, and 0.9%) with or without added MSG (0.3%) among 584 participants. The significant differences between each column are indicated by alphabetic superscripts. A column is significantly different from others that do not have the same superscript according to Tukey’s test (*P* < 0.05). An asterisk represents *P* for repeated measures ANOVA

The saltiness VAS ratings of the six samples are shown in Fig. 2A. The saltiness rating gradually increased in a manner dependent on the NaCl concentration of the test samples (0.3, 0.6, and 0.9%). The perceived saltiness of the 0.3% NaCl solution with MSG was significantly higher than that of the 0.3% NaCl without MSG (39.1 ± 0.7 versus 23.5 ± 0.6, *p* < 0.0001). The 0.6% NaCl with MSG solution was rated as slightly saltier than the 0.6% NaCl without MSG solution (58.2 ± 0.7 versus 55.8 ± 0.7, *p* = 0.0070). However, no significant difference in the rating of saltiness was observed between the 0.9% NaCl with MSG solution and the 0.9% NaCl without MSG solution (72.3 ± 0.7 versus 73.8 ± 0.6, *p* = 0.3013).

The mean and SE of saltiness VAS ratings are shown in Table 2 by sex, age group, smoking, fasting status, and medication status. Irrespective of subject characteristics such as sex, age, smoking experience and so on, the intensity of saltiness was dependent on the NaCl concentration of the test samples. In all groups, the saltiness VAS ratings of the 0.3% NaCl with MSG solution were significantly higher than those of the 0.3% NaCl without MSG solution, but for the 0.9% NaCl solution, there were no differences in saltiness ratings between the solution with and without MSG. However, for the 0.6% NaCl solution, differences due to subject characteristics were observed. That is, there was no significant difference in saltiness detected by males, individuals 30 years old or older, smokers, past smokers, individuals within 2 h after eating, or medicine users, whereas the addition of MSG significantly enhanced the saltiness detected by women, individuals under the age of 30, nonsmokers, and medicine nonusers.

**Table 2:** Mean and SE of saltiness VAS ratings by age, sex, smoking, fasting time, and medication

		0.3% NaCl		0.6% NaCl		0.9% NaCl		*P* value*
	N	−MSG	+MSG	−MSG	+MSG	−MSG	+MSG	
All	584	23.5 ± 0.6^a^	39.1 ± 0.7^b^	55.8 ± 0.7^c^	58.2 ± 0.7^d^	73.8 ± 0.6^e^	72.3 ± 0.7^e^	<0.0001
Sex								
Male	69	22.1 ± 1.7^a^	34.6 ± 2.0^b^	49.6 ± 2.3^c^	52.9 ± 2.0^c^	70.0 ± 1.8^d^	67.2 ± 1.9^d^	<0.0001
Female	515	23.7 ± 0.7^a^	39.7 ± 0.8^b^	56.6 ± 0.7^c^	58.9 ± 0.7^d^	74.3 ± 0.7^e^	73.0 ± 0.7^e^	<0.0001
Age group (year)								
19–29	257	24.0 ± 0.9^a^	45.3 ± 1.0^b^	61.2 ± 0.9^c^	65.6 ± 0.8^d^	79.0 ± 0.8^e^	79.5 ± 0.7^e^	<0.0001
30–59	72	21.4 ± 1.8^a^	35.0 ± 2.1^b^	51.2 ± 2.2^c^	56.7 ± 2.0^c^	71.2 ± 2.1^d^	69.1 ± 2.2^d^	<0.0001
60–69	100	21.8 ± 1.5^a^	34.2 ± 1.7^b^	52.7 ± 1.7^c^	52.5 ± 1.7^c^	69.8 ± 1.5^d^	66.8 ± 1.5^d^	<0.0001
>70	153	25.1 ± 1.2^a^	33.7 ± 1.3^b^	51.0 ± 1.4^c^	50.4 ± 1.5^c^	69.0 ± 1.3^d^	65.6 ± 1.4^d^	<0.0001
Smoking								
Smoker	14	19.6 ± 4.2^a^	36.8 ± 4.2^b^	54.6 ± 3.9^c^	51.0 ± 4.9^c^	78.4 ± 4.4^d^	70.9 ± 4.9^d^	<0.0001
Past smoke	28	24.8 ± 3.1^a^	34.5 ± 3.0^b^	49.2 ± 3.4^c^	55.1 ± 3.3^c^	72.3 ± 2.3^d^	65.6 ± 3.2^d^	<0.0001
Never smoker	539	23.6 ± 0.6^a^	39.4 ± 0.7^b^	56.2 ± 0.7^c^	58.6 ± 0.7^d^	73.8 ± 0.7^e^	72.7 ± 0.7^e^	<0.0001
Fasting time								
≤2 h	129	25.1 ± 1.4^a^	35.9 ± 1.5^b^	52.0 ± 1.7^c^	53.5 ± 1.5^c^	70.2 ± 1.5^d^	67.7 ± 1.4^d^	<0.0001
>2 h	453	23.0 ± 0.7^a^	39.9 ± 0.8^b^	56.8 ± 0.8^c^	59.6 ± 0.8^d^	74.8 ± 0.7^e^	73.7 ± 0.7^e^	<0.0001
Medicine								
With medication	199	24.2 ± 1.1^a^	36.1 ± 1.2^b^	53.6 ± 1.2^c^	54.2 ± 1.3^c^	70.6 ± 1.1^d^	68.3 ± 1.1^d^	<0.0001
No medication	382	23.3 ± 0.8^a^	40.8 ± 0.9^b^	57.0 ± 0.9^c^	60.5 ± 0.8^d^	75.5 ± 0.8^e^	74.6 ± 0.8^e^	<0.0001

The significant differences between each rating score are indicated by alphabetic superscripts. A rating score is significantly different from others that do not have the same superscript letter according to Tukey’s test (*P* < 0.05)

*P for repeated measures ANOVA

The saltiness VAS ratings are shown graphically in Supplementary Fig. 1A for the university participants and in Supplementary Fig. 2A for the health seminar attendees. Although there were some statistically significant differences, enhancement of saltiness by MSG was observed with the 0.3% NaCl solution irrespective of the region.

### Palatability

The palatability VAS ratings of 584 participants are shown in Fig. 2B. The palatability ratings of the test samples with MSG were significantly higher than those without MSG for all NaCl concentrations. In particular, the palatability rating of the 0.3% NaCl with MSG solution was almost twice that of the 0.3% NaCl solution without MSG (60.6 ± 0.9 versus 30.7 ± 0.8, *p* < 0.0001). The palatability rating was significantly higher among the solutions in the following order: 0.3% NaCl with MSG (60.6 ± 0.9), 0.6% NaCl with MSG (57.4 ± 0.7), 0.9% with MSG (51.6 ± 0.8), 0.6% NaCl without MSG (44.8 ± 0.7), 0.9% NaCl without MSG (40.7 ± 0.7), and 0.3% NaCl without MSG (30.7 ± 0.8).

The mean and SE of the palatability VAS ratings are shown in Table 3 by sex, age group, smoking, fasting, and medication status. The palatability was rated similarly and was significantly higher for the solutions with MSG than for those without MSG in all samples containing 0.3, 0.6, and 0.9% NaCl. Regardless of the NaCl concentration of the test solutions, MSG significantly enhanced their palatability. In all groups, the palatability ratings of the 0.3% NaCl solution with MSG were almost twice as high as those of the 0.3% NaCl solution without MSG and were highest among the six samples. Although there were some statistically significant differences, palatability was rated similarly higher for the solutions with MSG than those without MSG in all samples of 0.3, 0.6, and 0.9% NaCl, irrespective of the subjects’ characteristics.

**Table 3:** Mean and SE of palatability VAS ratings by age, sex, smoking, fasting time, and medication

		0.3% NaCl		0.6% NaCl		0.9% NaCl		*P* value*
	N	−MSG	+MSG	−MSG	+MSG	−MSG	+MSG	
All	584	30.7 ± 0.8^a^	60.6 ± 0.9^b^	44.8 ± 0.7^c^	57.4 ± 0.7^d^	40.7 ± 0.7^e^	51.6 ± 0.8^f^	<0.0001
Sex								
Male	69	28.9 ± 2.2^a^	56.2 ± 2.6^b^	39.7 ± 1.9^c^	54.1 ± 1.7^b^	41.6 ± 2.0^c^	50.3 ± 2.3^b^	<0.0001
Female	515	30.9 ± 0.8^a^	61.2 ± 1.0^b^	45.4 ± 0.7^c^	57.8 ± 0.8^d^	40.5 ± 0.8^e^	51.7 ± 0.9^f^	<0.0001
Age group (year)								
19–29	257	32.1 ± 1.2^a^	61.6 ± 1.4^b^	47.4 ± 1.0^c^	58.8 ± 1.1^b^	42.4 ± 1.1^d^	51.7 ± 1.3^c^	<0.0001
30–59	72	25.9 ± 1.8^a^	65.5 ± 2.7^b^	43.5 ± 2.3^c,d^	57.1 ± 2.2^e^	38.9 ± 2.1^c^	51.5 ± 2.5^d,e^	<0.0001
60–69	100	27.8 ± 1.8^a^	59.5 ± 2.0^b^	42.3 ± 1.6^c^	55.1 ± 1.7^b,d^	39.9 ± 1.7^c^	51.5 ± 1.8^d^	<0.0001
>70	153	32.6 ± 1.5^a^	57.8 ± 1.8^b^	42.6 ± 1.4^c^	56.6 ± 1.4^b,d^	39.1 ± 1.6^c^	51.6 ± 1.5^d^	<0.0001
Smoking								
Smoker	14	33.3 ± 5.9^a^	63.4 ± 4.5^b^	49.4 ± 5.4	62.0 ± 4.0^b^	40.4 ± 7.1^a,c^	55.3 ± 3.9^b,c^	0.0002
Past smoker	28	29.3 ± 3.5^a^	54.3 ± 4.2^b^	39.2 ± 2.8^a,c^	53.8 ± 3.2^b^	38.9 ± 2.9^a,c^	50.6 ± 3.9^b,c^	<0.0001
Never smoker	539	30.6 ± 0.8^a^	60.9 ± 0.9^b^	44.9 ± 0.7^c^	57.5 ± 0.8^d^	40.8 ± 0.8^e^	51.5 ± 0.9^f^	<0.0001
Fasting time								
≤2 h	129	33.3 ± 1.7^a^	59.7 ± 2.0^b^	43.1 ± 1.7^c^	57.3 ± 1.5^b^	37.5 ± 1.6^c^	49.8 ± 1.7^d^	<0.0001
>2 h	453	29.8 ± 0.8^a^	60.9 ± 1.0^b^	45.2 ± 0.8^c^	57.4 ± 0.8^d^	41.5 ± 0.8^e^	52.0 ± 0.9^f^	<0.0001
Medicine								
With medication	199	31.1 ± 1.3^a^	56.7 ± 1.5^b^	43.5 ± 1.2^c^	55.0 ± 1.2^b,d^	41.2 ± 1.3^c^	50.5 ± 1.3^d^	<0.0001
No medication	382	30.6 ± 0.9^a^	62.9 ± 1.1^b^	45.5 ± 0.9^c^	58.7 ± 0.9^d^	40.4 ± 0.9^e^	52.1 ± 1.1^f^	<0.0001

The significant differences between each rating score are indicated by alphabetic superscripts. A rating score is significantly different from others that have different superscript letters according to Tukey’s test (*P* < 0.05). A rating score without any alphabetic superscripts is not significantly different from any other score

^*^P for repeated-measures ANOVA

The palatability VAS ratings are shown graphically in Supplementary Fig. 1B for the university participants and in Supplementary Fig. 2B for the health seminar attendees. Although there were some statistically significant differences, the palatability was rated similarly higher for the solutions with MSG than for those without MSG in all samples of 0.3, 0.6, and 0.9% NaCl, irrespective of the region.

## Discussion

This study demonstrates that umami (MSG) increases the palatability of low-salt solutions irrespective of subject characteristics such as sex, age, region and status of smoking, fasting or medication. A higher enhancement effect of MSG on palatability was observed at lower salt concentrations, and a marked effect on the 0.3% NaCl solution was found.

These results provide support for the effect of MSG on food palatability [29–36, 39–41]. In previous studies investigating the interaction of NaCl and umami (MSG or CDG) in different types of soups, it was shown that the addition of umami allows considerable salt reductions in soup without affecting the pleasantness, saltiness, or taste intensity [29–35]. However, the optimal levels of NaCl and MSG (or CDG) in the soup were different in each experiment. For example, a combination of 0.3% MSG and 0.7% NaCl was shown to be most suitable for spicy soups [35]. In addition, clear soup with 0.38% MSG and 0.81% NaCl was more palatable [29]. Carter et al. suggested that CDG could partly replace NaCl and that chicken broth with 0.17% CDG and 0.85% NaCl had high likeability and pleasantness ratings [34]. These previous studies used much more complex soups that contained ingredients such as spices, dried bonito, chicken extract, pumpkin, mushroom, lentil, leek-potato, various vegetables [29–35], and other foods (beef jelly, spinach) [36]. These ingredients should be considered to affect the perception of taste, intensity of saltiness and palatability of the soup or food.

The current study used tasteless and odorless distilled water as a medium, and based on preliminary experiments (unpublished data), test solutions with different levels of NaCl and MSG were prepared. The participants in this sensory evaluation experiment included general adults in multiple regions of Japan who tasted all test solutions twice in a double-blind randomized manner under similar circumstances. Therefore, our results can help generalize previous findings. The enhancement of palatability by adding MSG was observed at any concentration of NaCl, and the palatability of the 0.3% NaCl solution showed the strongest enhancement. However, the saltiness of the 0.9% NaCl solution was not significantly enhanced by adding MSG. These results indicate that palatability enhancement due to the addition of MSG could not be explained only by the enhancement of saltiness due to the addition of MSG. Although it should be considered that the complexity of taste perception in the brain may affect sensory evaluation, it is likely that MSG has the capability to compensate for the loss of palatability caused by salt reduction. Thus, the NaCl concentration can be reduced from 0.9 to 0.3% while maintaining meal satisfaction. The sodium concentration of the 0.3% NaCl solution with MSG was 0.155 g/ml and corresponded to 36.9% of the sodium content of the 0.9% NaCl solution with MSG (Table 4). This modification of the solution leads to a 60.4% reduction in sodium intake. As mentioned above, in the preliminary study, 0.6% NaCl and 0.3% MSG solution showed the highest palatability. However, a previous study aimed to determine the applicable concentrations of NaCl and MSG. The sensory evaluation test was conducted for 6 days, and the participants evaluated one series of NaCl/MSG mixtures with one MSG concentration. In addition, the test solutions were provided in order from lowest to highest NaCl concentrations. Hence, these differences in the protocols are thought to have brought about the discrepancy between the results of the previous and current studies.

**Table 4:** Sodium (Na) content of the six test solutions

NaCl (g/100 mL)	0.3		0.6		0.9	
0.3% MSG	–	+	–	+	–	+
Na (g/100 mL)	0.118	0.155	0.236	0.273	0.354	0.391
Na (100%: 0.9% NaCl + 0.3% MSG)^a^	30.2	39.6	60.4	69.8	90.5	100

^a^Assuming sodium content to be 100% in the sample containing 0.9% NaCl with 0.3% MSG, the amounts of sodium in the other samples are indicated

Bruins et al. estimated the public health impact of soups containing ~25% less sodium and concluded that reduced-salt soups may be beneficial for reducing cardiovascular disease and mortality [40]. Moreover, Webb et al. reported that a government “soft regulation” strategy combining targeted industry agreements and public education to reduce dietary sodium is projected to be highly cost effective worldwide, even without accounting for potential healthcare savings [41].

Regardless of sex, age, region, smoking experience, fasting or dosing status, significant saltiness enhancement by MSG was observed with the 0.3% NaCl solution, but no significant difference was observed with the 0.9% NaCl solution in the subanalysis. On the other hand, for the 0.6% NaCl solution, significant differences that were dependent on subject characteristics were observed. In some cases, the number of participants for the subanalysis was insufficient. Hence, further analysis is needed to draw conclusions. Concerning palatability, increases in its ratings were observed with the addition of MSG in the 0.3, 0.6, and 0.9% NaCl solutions, regardless of sex, age, and fasting or medication status. The importance of MSG enhancement for the 0.6% and 0.9% NaCl solutions was not observed in smokers or former smokers or in some regions, but this finding was influenced by the small number of participants. The significant improvement in palatability when MSG was added to the 0.3% NaCl solution is likely to be the result of evaluation across differences in sex, age, place of residence, smoking, fasting, or medication status.

It was demonstrated that the preferred level of salt depended on habitual dietary levels [41, 42] and that this preferred level could be reduced after a reduction in sodium intake for several months [43]. In a longitudinal study, Blais et al. found that subjects adapted to the taste of foods containing less salt after dietary restriction of their sodium intake [42]. Moreover, it is generally believed that the optimal salt concentration is dependent on daily salt intake, which varies in each region in Japan. However, no significant differences in saltiness or palatability were found among any regions. In this analysis, the number of participants in each region was limited, and detailed eating habits were not surveyed; thus, we could not exclude the bias that occurred in daily salt intake, food preference or preferred salt intensity. The influence of regional eating habits and individual daily salt intake should be further studied.

The present study has several limitations. First, in this study, the total subject group was composed of male and female students and health seminar attendees. There were many sex and age biases, many of the participants had high health awareness, and the effect of selection bias should be considered. The subjects seem to prefer less salt in their daily meals than the general population. However, the subjects were free-living adult men and women in multiple regions in Japan and had no experience in taste training. Since there are no specific target characteristics or regional characteristics, the results of this study may be applied to the general population as it is. Second, the subjects of this study were untrained people. However, Sinesio et al. showed that untrained subjects could distinguish between soups with increasing umami flavor, even though they could not identify the specific attribute umami [38]. Finally, as we did not collect information such as height, weight, and blood pressure, we cannot examine relationships with individual characteristics such as physical condition.

The limitations described above suggest that further study of the effect of MSG may be needed. However, the characteristics of the participants in this study are not so different from those of the average person. Consequently, our results obtained in this study suggest the possibility of reducing the salt concentration of clear soup to 0.3% from 0.9% or 0.6% without a loss of palatability by adding 0.3% MSG, which leads to a 60.4% reduction in sodium intake. It is better to add MSG to low-salt soup for a healthy and delicious dietary lifestyle. Utilizing an appropriate amount of umami in soup would contribute to improved human health.

## Supplementary information

Supplementary Figure

## References

[1] Turnbull F (2005). Managing cardiovascular risk factors: the gap between evidence and practice. PLoS Med.

[2] Strazzullo P, D Elia L, Kandala NB, Kandala NB, Cappuccio FP (2009). Salt intake, stroke, and cardiovascular disease: meta-analysis of prospective studies. BMJ.

[3] Aburto NJ, Ziolkovska A, Hooper L, Elliott P, Cappiccio FP, Meerpohl JJ (2013). Effect of lower sodium intake on health: systematic review and meta-analyses. BMJ.

[4] Cook NR, Appel LJ, Whelton PK (2014). Lower levels of sodium intake and reduced cardiovascular risk. Circulation.

[5] Tuomilehto J, Jousilahti P, Rastenyte D, Moltchanov V, Tanskanen A, Pietinen P (2001). Urinary sodium excretion and cardiovascular mortality in Finland: a prospective study. Lancet..

[6] Cook NR, Appel LJ, Whelton PK (2016). Sodium intake and all-cause mortality over 20 years in the trials of hypertension prevention. J Am Coll Cardiol.

[7] Liu H, Gao X, Zho L, Wu Y, Li Y, Mai J (2018). Urinary sodium excretion and risk of cardiovascular disease in the Chinese population: a prospective study. Hypertens Res.

[8] Dahl LK, Love RA (1954). Evidence for relationship between sodium (chloride) intake and human essential hypertension. AMA Arch Intern Med.

[9] Intersalt Cooperative Research Group. (1988). Intersalt: an international study of electrolyte excretion and blood pressure: results for 24 h urinary sodium and potassium excretion. BMJ.

[10] Mente A, O’Donnell MJ, Rangarajan S, McQueen MJ, Poirier P, Wielgosz A (2014). Association of urinary sodium and potassium excretion with blood pressure. N Eng J Med.

[11] Chan Q, Stamler J, Griep LMO, Daviglus ML, Horn LV, Elliott P (2016). An update on nutrients and blood pressure. J Atheroscler Thromb.

[12] Stamler J, Chan Q, Daviglus ML, Dyer AR, Van Horn L, Garside DB (2018). Relation of dietary sodium (salt) to blood pressure and its possible modulation by other dietary factors: the INTERMA Study. Hypertension.

[13] Lim SS, Vos T, Flaxman AD, Danaei G, Shibuya K, Rohani HA (2012). A comparative risk assessment of burden of disease and injury attributable to 67 risk factors and risk factor clusters in 21 regions, 1990-2010: a systematic analysis for the Global Burden of Disease Study 2010. Lancet.

[14] Kearney PM, Whelton M, Reynolds K, Muntner P, Whelton PK, He J (2005). Global burden of hypertension: analysis of worldwide data. Lancet.

[15] Poulter NR, Prabhakaran D, Caulfield M (2015). Hypertension. Lancet.

[16] Umemura S, Arima H, Arima S, Asayama K, Dohi Y, Yoshitake H (2019). The Japanese Society of Hypertension Guidelines for the Management of Hypertension (JSH2019). Hypertens Res.

[17] Ministry of Health, Labour and Welfare. The National Health and Nutrition Survey Japan. Japan: Ministry of Health, Labour and Welfare; 2017. https://www.mhlw.go.jp/content/10904750/000351576.pdf. Accessed 10 July 2019.

[18] Ministry of Health, Labour and Welfare, Japan. Dietary reference intakes for Japanese, 2015. Tokyo, Japan: Daiichi-syuppan; 2015.

[19] World Health Organization (WHO). Guideline: sodium intake for adults and children. https://apps.who.int/iris/handle/10665/77985. Accessed 10 July 2019. 23658998

[20] World Health Organization (WHO). Global action plan for the prevention and control of non-communicable diseases 2013-220. https://www.who.int/nmh/events/ncd_action_plan/en/. Accessed 12 July 2019.

[21] Trieu K, Neal B, Hawkes C, Dunford E, Campbell N, Rodriguez FR (2015). Salt reduction initiatives around the world—A systematic review of progress towards the global target. PLoS ONE.

[22] Mozaffarian D, Fahimi S, Singh GM, Micha R, Khatibzadeh S, Engell RE (2014). Global sodium consumption and death from cardiovascular causes. NEJM.

[23] He FJ, Li J, MacGregor GA (2013). Effect of longer term modest salt reduction on blood pressure: cochrane systematic review and meta-analysis of randomised trials. BMJ.

[24] Hoppu U, Hopia A, Pohjanheimo T, Rotola-Pukkila M, Mäkinen S, Pihlanto A, Sandell M (2017). Effect of salt reduction on consumer acceptance and aensory quality of food. Foods.

[25] Breslin PAS, Beauchamp GK (1997). Salt enhances flavour by suppressing bitterness. Nature.

[26] Okuda N, Stamler J, Browne IJ, Ueshima H, Miura K, Okayama A (2014). Individual efforts to reduce salt intake in China, Japan, UK, USA: what did people achieve? The INTERMAP Population Study. J Hypertens.

[27] Kawamura A, Kajiya K, Kishi H, Inagaki J, Mitarai M, Oda H (2016). Effects of the DASH-JUMP dietary interventional Japanese participants with high-normal blood pressure and stage 1 hypertension: an open-label single -arm trial. Hypertens Res.

[28] Kubota Y, Higashiyama A, Sugiyama D, Nishida Y, Kubo S, Hirata T (2018). Association between impairment of salty taste recognition and masked hypertension based on home blood pressure in Japanese resident: the KOBE study. Hypertens Res.

[29] Yamaguchi S, Takahashi C (1984). Interactions of monosodium glutamate and sodium chloride on saltiness and palatability of a clear soup. J Food Sci.

[30] Daget N, Guion P (1989). Influence of glutamic acid or its salts on the sensory characteristics of a chicken broth: reduction of sodium intake. Food Qual Prefer.

[31] Roininen K, Lahteemak L, Tuorile H (1996). Effect of umami taste on pleasantness of low-salt soups during repeated testing. Physiol Behav.

[32] Okiyama A, Beauchamp GK (1998). Taste dimensions of monosodium glutamate (MSG) in a food system: role of glutamate in young American subjects. Physiol Behav.

[33] Ball P, Woodward D, Beard T, Shoobridge A, Ferrier M (2002). Calcium diglutamate improves taste characteristics of lower-salt soup. Eur J Clin Nutr.

[34] Carter BE, Monsivais P, Drewnowski A (2011). The sensory optimum of chicken broths supplemented with calcium di-glutamate: a possibility for reducing sodium while maintaining taste. Food Qual Prefer.

[35] Jinap S, Hajeb P, Karim R, Norliana S, Yibadatihan S, Abdul-Kadir R (2016). Reduction of sodium content in spicy soups using monosodium glutamate. Food Nutr Res.

[36] Bellisle F, Tournier A, Louis-Sylvestre J (1989). Monosodium glutamate and the acquisition of food preferences in a European context. Food Qual Prefer.

[37] Takimoto H, Saito A, Htun NC, Abe K (2018). Food items contributing to high dietary salt intake among Japanese adults in the 2012 National Health and Nutrition Survey. Hypertens Res.

[38] Flint A, Raven A, Blundell J, Astrup A (2000). Reproducibility, power and validity of visual analogue scales in assessment of appetite sensations in single test meal studies. Int J Obes Relat Metab Disord.

[39] Sinesio F, Peparaio M, Moneta E, Comendador FJ (2010). Perceptive maps of dishes varying in glutamate content with professional and naive subjects. Food Qual Prefer.

[40] Bruins MJ, Dötsch-Klerk M, Matthee J, Kearney M, van Elk K, Weber P, Eggersdorfer M (2015). A modelling approach to estimate the impact of sodium reduction in soups on cardiovascular health in the Netherlands. Nutrients.

[41] Webb M, Fahimi S, Singh GM, Khatibzadeh S, Micha R, Powles J, Mozaffarian D (2017). Cost effectiveness of a government supported policy strategy to decrease sodium intake: global analysis across 183 nations. BMJ.

[42] Blais CA, Pangbom RS, Borhani NO, Ferrell MF, Prineas RJ, Laing B (1986). Effect of dietary sodium restriction on taste responses to sodium chloride: a longitudinal study. Am Clin Nutr.

[43] Bertino M, Beauchamp GK, Engelman K (1982). Long term reduction in dietary sodium alter the taste of salt. Am J Clin Nutr.

