# Supplementary material for: Validation of preferred salt concentration in soup based on a randomized blinded experiment in multiple regions in Japan—influence of umami (l-glutamate) on saltiness and palatability of low-salt solutions
Source: Hypertens Res. 2020 Jan 29;43(6):525–33. doi: 10.1038/s41440-020-0397-1 (PMC8075858; doi:10.1038/s41440-020-0397-1)
Supplement: Supplementary file 1 — Supplementary Figure [file 41440_2020_397_MOESM1_ESM.pptx]

## Slide 1
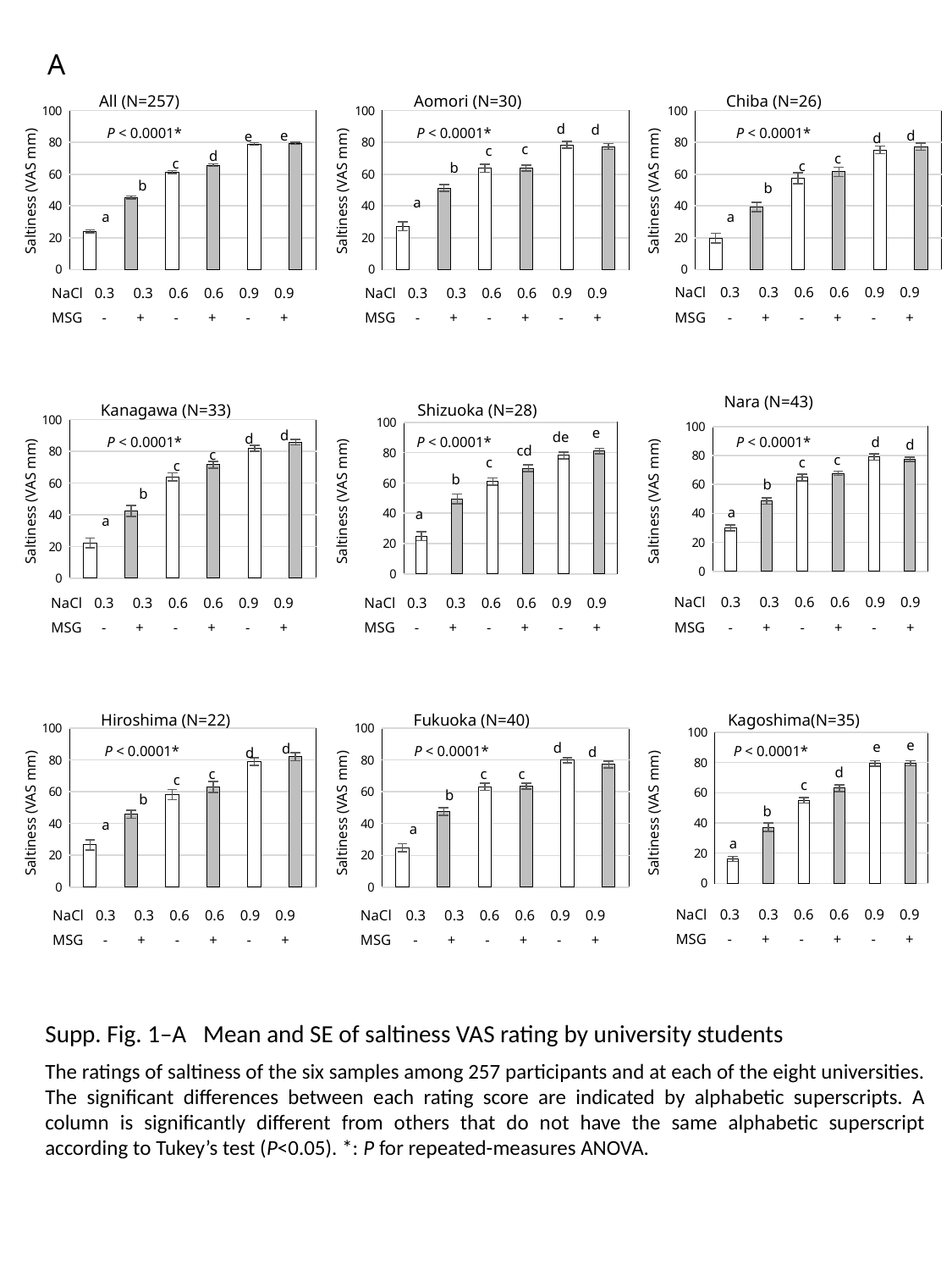

A
All (N=257)
Aomori (N=30)
Chiba (N=26)
### Chart
| Category | |
|---|---|
| NaCl0.3MSG0 | 24.080739299610894 |
| NaCl0.3MSG0.3 | 45.311284046692606 |
| NaCl0.6MSG0 | 61.262645914396884 |
| NaCl0.6MSG0.3 | 65.64396887159533 |
| NaCl0.9MSG0 | 79.02529182879377 |
| NaCl0.9MSG0.3 | 79.52431906614785 |
### Chart
| Category | |
|---|---|
| NaCl0.3MSG0 | 27.258333333333333 |
| NaCl0.3MSG0.3 | 51.333333333333336 |
| NaCl0.6MSG0 | 63.75 |
| NaCl0.6MSG0.3 | 63.86666666666667 |
| NaCl0.9MSG0 | 78.43333333333334 |
| NaCl0.9MSG0.3 | 77.41666666666667 |
### Chart
| Category | |
|---|---|
| NaCl0.3MSG0 | 19.78846153846154 |
| NaCl0.3MSG0.3 | 39.40384615384615 |
| NaCl0.6MSG0 | 57.46153846153846 |
| NaCl0.6MSG0.3 | 61.46153846153846 |
| NaCl0.9MSG0 | 75.25961538461539 |
| NaCl0.9MSG0.3 | 77.32692307692308 |d
d
d
e
e
d
c
c
d
c
c
c
b
b
b
Saltiness (VAS mm)
Saltiness (VAS mm)
Saltiness (VAS mm)
a
a
a
NaCl
0.3
0.3
0.6
0.6
0.9
0.9
NaCl
0.3
0.3
0.6
0.6
0.9
0.9
NaCl
0.3
0.3
0.6
0.6
0.9
0.9
MSG
-
+
-
+
-
+
MSG
-
+
-
+
-
+
MSG
-
+
-
+
-
+
Nara (N=43)
Shizuoka (N=28)
Kanagawa (N=33)
### Chart
| Category | |
|---|---|
| NaCl0.3MSG0 | 22.12878787878788 |
| NaCl0.3MSG0.3 | 42.378787878787875 |
| NaCl0.6MSG0 | 63.84090909090909 |
| NaCl0.6MSG0.3 | 71.50757575757575 |
| NaCl0.9MSG0 | 81.81060606060606 |
| NaCl0.9MSG0.3 | 85.74242424242425 |
### Chart
| Category | |
|---|---|
| NaCl0.3MSG0 | 25.0 |
| NaCl0.3MSG0.3 | 49.4375 |
| NaCl0.6MSG0 | 60.919642857142854 |
| NaCl0.6MSG0.3 | 69.72321428571429 |
| NaCl0.9MSG0 | 78.25 |
| NaCl0.9MSG0.3 | 80.98214285714286 |
### Chart
| Category | |
|---|---|
| NaCl0.3MSG0 | 29.912790697674417 |
| NaCl0.3MSG0.3 | 48.616279069767444 |
| NaCl0.6MSG0 | 64.84302325581395 |
| NaCl0.6MSG0.3 | 67.68604651162791 |
| NaCl0.9MSG0 | 78.99418604651163 |
| NaCl0.9MSG0.3 | 77.43023255813954 |e
d
de
d
d
d
cd
c
c
c
c
c
b
b
b
Saltiness (VAS mm)
Saltiness (VAS mm)
Saltiness (VAS mm)
a
a
a
NaCl
0.3
0.3
0.6
0.6
0.9
0.9
NaCl
0.3
0.3
0.6
0.6
0.9
0.9
NaCl
0.3
0.3
0.6
0.6
0.9
0.9
MSG
-
+
-
+
-
+
MSG
-
+
-
+
-
+
MSG
-
+
-
+
-
+
Hiroshima (N=22)
Fukuoka (N=40)
Kagoshima(N=35)
### Chart
| Category | |
|---|---|
| NaCl0.3MSG0 | 26.59090909090909 |
| NaCl0.3MSG0.3 | 45.79545454545455 |
| NaCl0.6MSG0 | 58.19318181818182 |
| NaCl0.6MSG0.3 | 62.96590909090909 |
| NaCl0.9MSG0 | 79.07954545454545 |
| NaCl0.9MSG0.3 | 82.04545454545455 |
### Chart
| Category | |
|---|---|
| NaCl0.3MSG0 | 24.79375 |
| NaCl0.3MSG0.3 | 47.4625 |
| NaCl0.6MSG0 | 63.21875 |
| NaCl0.6MSG0.3 | 63.58125 |
| NaCl0.9MSG0 | 79.825 |
| NaCl0.9MSG0.3 | 77.14375 |
### Chart
| Category | |
|---|---|
| NaCl0.3MSG0 | 16.09285714285714 |
| NaCl0.3MSG0.3 | 37.17857142857143 |
| NaCl0.6MSG0 | 55.09285714285714 |
| NaCl0.6MSG0.3 | 63.01428571428571 |
| NaCl0.9MSG0 | 79.41428571428571 |
| NaCl0.9MSG0.3 | 79.64285714285714 |e
e
d
d
d
d
d
c
c
c
c
c
b
b
b
Saltiness (VAS mm)
Saltiness (VAS mm)
Saltiness (VAS mm)
a
a
a
NaCl
0.3
0.3
0.6
0.6
0.9
0.9
NaCl
0.3
0.3
0.6
0.6
0.9
0.9
NaCl
0.3
0.3
0.6
0.6
0.9
0.9
MSG
-
+
-
+
-
+
MSG
-
+
-
+
-
+
MSG
-
+
-
+
-
+
P < 0.0001*
P < 0.0001*
P < 0.0001*
P < 0.0001*
P < 0.0001*
P < 0.0001*
P < 0.0001*
P < 0.0001*
P < 0.0001*
Supp. Fig. 1–A Mean and SE of saltiness VAS rating by university students
The ratings of saltiness of the six samples among 257 participants and at each of the eight universities. The significant differences between each rating score are indicated by alphabetic superscripts. A column is significantly different from others that do not have the same alphabetic superscript according to Tukey’s test (P<0.05). *: P for repeated-measures ANOVA.

## Slide 2
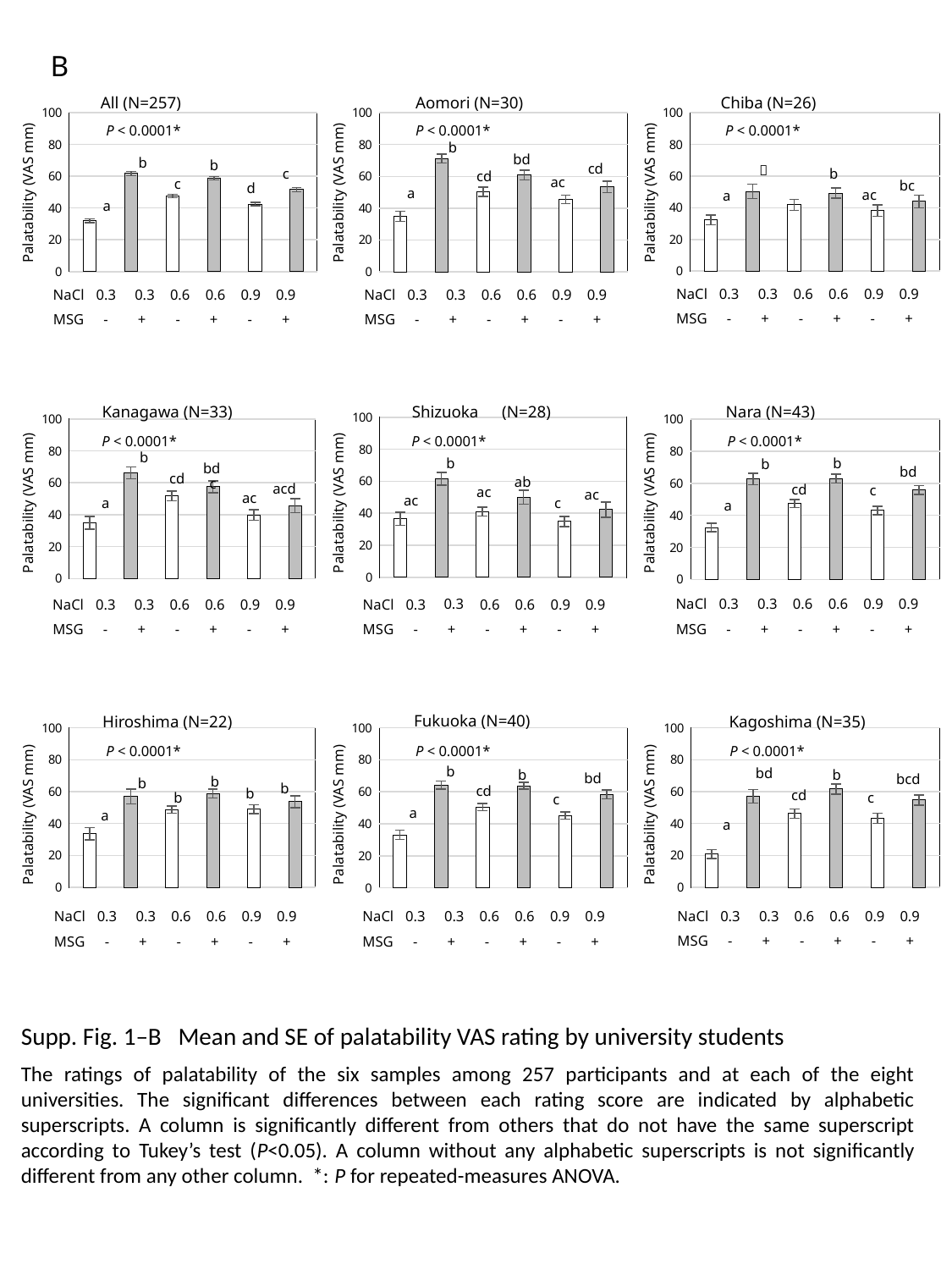

B
All (N=257)
Aomori (N=30)
Chiba (N=26)
### Chart
| Category | |
|---|---|
| NaCl0.3MSG0 | 32.07587548638132 |
| NaCl0.3MSG0.3 | 61.71692607003891 |
| NaCl0.6MSG0 | 47.46887159533074 |
| NaCl0.6MSG0.3 | 58.78307392996109 |
| NaCl0.9MSG0 | 42.41050583657587 |
| NaCl0.9MSG0.3 | 51.62451361867704 |
### Chart
| Category | |
|---|---|
| NaCl0.3MSG0 | 34.93333333333333 |
| NaCl0.3MSG0.3 | 71.25833333333334 |
| NaCl0.6MSG0 | 50.325 |
| NaCl0.6MSG0.3 | 60.725 |
| NaCl0.9MSG0 | 45.55 |
| NaCl0.9MSG0.3 | 53.30833333333333 |
### Chart
| Category | |
|---|---|
| NaCl0.3MSG0 | 32.28846153846154 |
| NaCl0.3MSG0.3 | 50.25 |
| NaCl0.6MSG0 | 41.84615384615385 |
| NaCl0.6MSG0.3 | 49.18269230769231 |
| NaCl0.9MSG0 | 38.19230769230769 |
| NaCl0.9MSG0.3 | 43.96153846153846 |b
bd
b
b
cd
ｂ
c
b
cd
ac
c
bc
d
Palatability (VAS mm)
Palatability (VAS mm)
Palatability (VAS mm)
a
ac
a
a
NaCl
0.3
0.3
0.6
0.6
0.9
0.9
NaCl
0.3
0.3
0.6
0.6
0.9
0.9
NaCl
0.3
0.3
0.6
0.6
0.9
0.9
MSG
-
+
-
+
-
+
MSG
-
+
-
+
-
+
MSG
-
+
-
+
-
+
Shizuoka　(N=28)
Kanagawa (N=33)
Nara (N=43)
### Chart
| Category | |
|---|---|
| NaCl0.3MSG0 | 36.5625 |
| NaCl0.3MSG0.3 | 61.482142857142854 |
| NaCl0.6MSG0 | 41.107142857142854 |
| NaCl0.6MSG0.3 | 50.160714285714285 |
| NaCl0.9MSG0 | 34.88392857142857 |
| NaCl0.9MSG0.3 | 42.25892857142857 |
### Chart
| Category | |
|---|---|
| NaCl0.3MSG0 | 34.946969696969695 |
| NaCl0.3MSG0.3 | 66.1590909090909 |
| NaCl0.6MSG0 | 51.82575757575758 |
| NaCl0.6MSG0.3 | 57.54545454545455 |
| NaCl0.9MSG0 | 39.79545454545455 |
| NaCl0.9MSG0.3 | 45.621212121212125 |
### Chart
| Category | |
|---|---|
| NaCl0.3MSG0 | 32.395348837209305 |
| NaCl0.3MSG0.3 | 62.651162790697676 |
| NaCl0.6MSG0 | 47.28488372093023 |
| NaCl0.6MSG0.3 | 62.872093023255815 |
| NaCl0.9MSG0 | 42.97674418604651 |
| NaCl0.9MSG0.3 | 55.81395348837209 |b
b
b
b
bd
bd
cd
ab
c
acd
cd
c
ac
ac
ac
ac
a
c
Palatability (VAS mm)
Palatability (VAS mm)
Palatability (VAS mm)
a
0.3
NaCl
0.3
0.3
0.6
0.6
0.9
0.9
NaCl
0.3
0.3
0.6
0.6
0.9
0.9
NaCl
0.3
0.6
0.6
0.9
0.9
MSG
-
+
-
+
-
+
MSG
-
+
-
+
-
+
MSG
-
+
-
+
-
+
Fukuoka (N=40)
Hiroshima (N=22)
Kagoshima (N=35)
### Chart
| Category | |
|---|---|
| NaCl0.3MSG0 | 33.48863636363637 |
| NaCl0.3MSG0.3 | 56.89772727272727 |
| NaCl0.6MSG0 | 48.60227272727273 |
| NaCl0.6MSG0.3 | 58.76136363636363 |
| NaCl0.9MSG0 | 49.03409090909091 |
| NaCl0.9MSG0.3 | 53.76136363636363 |
### Chart
| Category | |
|---|---|
| NaCl0.3MSG0 | 33.00625 |
| NaCl0.3MSG0.3 | 64.2 |
| NaCl0.6MSG0 | 50.5 |
| NaCl0.6MSG0.3 | 63.7375 |
| NaCl0.9MSG0 | 45.21875 |
| NaCl0.9MSG0.3 | 58.425 |
### Chart
| Category | |
|---|---|
| NaCl0.3MSG0 | 20.82857142857143 |
| NaCl0.3MSG0.3 | 57.1 |
| NaCl0.6MSG0 | 46.22857142857143 |
| NaCl0.6MSG0.3 | 61.642857142857146 |
| NaCl0.9MSG0 | 43.27142857142857 |
| NaCl0.9MSG0.3 | 54.76428571428571 |b
bd
b
b
bd
bcd
b
b
b
cd
b
cd
c
b
c
a
Palatability (VAS mm)
Palatability (VAS mm)
Palatability (VAS mm)
a
a
NaCl
0.3
0.3
0.6
0.6
0.9
0.9
NaCl
0.3
0.3
0.6
0.6
0.9
0.9
NaCl
0.3
0.3
0.6
0.6
0.9
0.9
MSG
-
+
-
+
-
+
MSG
-
+
-
+
-
+
MSG
-
+
-
+
-
+
P < 0.0001*
P < 0.0001*
P < 0.0001*
P < 0.0001*
P < 0.0001*
P < 0.0001*
P < 0.0001*
P < 0.0001*
P < 0.0001*
Supp. Fig. 1–B Mean and SE of palatability VAS rating by university students
The ratings of palatability of the six samples among 257 participants and at each of the eight universities. The significant differences between each rating score are indicated by alphabetic superscripts. A column is significantly different from others that do not have the same superscript according to Tukey’s test (P<0.05). A column without any alphabetic superscripts is not significantly different from any other column. *: P for repeated-measures ANOVA.

## Slide 3
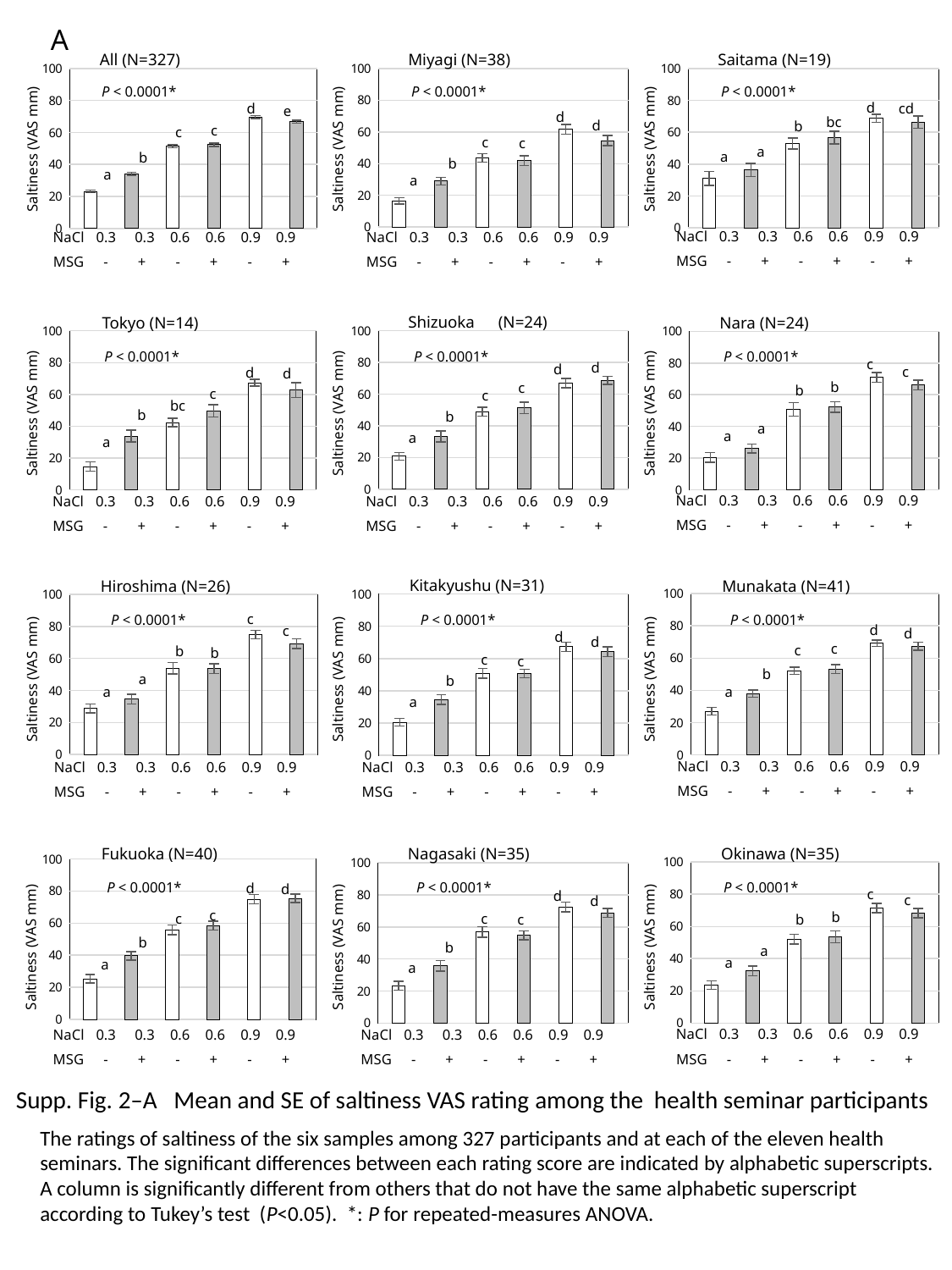

A
All (N=327)
Miyagi (N=38)
Saitama (N=19)
### Chart
| Category | |
|---|---|
| NaCl0.3MSG0 | 23.119266055045873 |
| NaCl0.3MSG0.3 | 34.17125382262997 |
| NaCl0.6MSG0 | 51.43042813455658 |
| NaCl0.6MSG0.3 | 52.37385321100918 |
| NaCl0.9MSG0 | 69.72018348623853 |
| NaCl0.9MSG0.3 | 66.7071865443425 |
### Chart
| Category | |
|---|---|
| NaCl0.3MSG0 | 16.57236842105263 |
| NaCl0.3MSG0.3 | 28.95394736842105 |
| NaCl0.6MSG0 | 43.61842105263158 |
| NaCl0.6MSG0.3 | 41.96052631578947 |
| NaCl0.9MSG0 | 61.56578947368421 |
| NaCl0.9MSG0.3 | 54.57236842105263 |
### Chart
| Category | |
|---|---|
| NaCl0.3MSG0 | 31.06578947368421 |
| NaCl0.3MSG0.3 | 36.28947368421053 |
| NaCl0.6MSG0 | 52.9078947368421 |
| NaCl0.6MSG0.3 | 56.68421052631579 |
| NaCl0.9MSG0 | 68.75 |
| NaCl0.9MSG0.3 | 66.35526315789474 |d
d
cd
e
d
bc
d
b
c
c
c
c
Saltiness (VAS mm)
Saltiness (VAS mm)
Saltiness (VAS mm)
a
a
b
b
a
a
NaCl
0.3
0.3
0.6
0.6
0.9
0.9
NaCl
0.3
0.3
0.6
0.6
0.9
0.9
NaCl
0.3
0.3
0.6
0.6
0.9
0.9
MSG
-
+
-
+
-
+
MSG
-
+
-
+
-
+
MSG
-
+
-
+
-
+
Shizuoka　(N=24)
Tokyo (N=14)
Nara (N=24)
### Chart
| Category | |
|---|---|
| NaCl0.3MSG0 | 14.625 |
| NaCl0.3MSG0.3 | 33.82142857142857 |
| NaCl0.6MSG0 | 42.32142857142857 |
| NaCl0.6MSG0.3 | 49.767857142857146 |
| NaCl0.9MSG0 | 67.375 |
| NaCl0.9MSG0.3 | 62.714285714285715 |
### Chart
| Category | |
|---|---|
| NaCl0.3MSG0 | 20.739583333333332 |
| NaCl0.3MSG0.3 | 33.177083333333336 |
| NaCl0.6MSG0 | 48.895833333333336 |
| NaCl0.6MSG0.3 | 51.260416666666664 |
| NaCl0.9MSG0 | 66.94791666666667 |
| NaCl0.9MSG0.3 | 68.63541666666667 |
### Chart
| Category | |
|---|---|
| NaCl0.3MSG0 | 20.5625 |
| NaCl0.3MSG0.3 | 26.03125 |
| NaCl0.6MSG0 | 50.8125 |
| NaCl0.6MSG0.3 | 52.239583333333336 |
| NaCl0.9MSG0 | 70.89583333333333 |
| NaCl0.9MSG0.3 | 66.08333333333333 |c
d
d
c
d
d
b
c
b
c
c
bc
Saltiness (VAS mm)
Saltiness (VAS mm)
Saltiness (VAS mm)
b
b
a
a
a
a
NaCl
0.3
0.3
0.6
0.6
0.9
0.9
NaCl
0.3
0.3
0.6
0.6
0.9
0.9
NaCl
0.3
0.3
0.6
0.6
0.9
0.9
MSG
-
+
-
+
-
+
MSG
-
+
-
+
-
+
MSG
-
+
-
+
-
+
Kitakyushu (N=31)
Hiroshima (N=26)
Munakata (N=41)
### Chart
| Category | |
|---|---|
| NaCl0.3MSG0 | 27.067073170731707 |
| NaCl0.3MSG0.3 | 37.93292682926829 |
| NaCl0.6MSG0 | 52.26829268292683 |
| NaCl0.6MSG0.3 | 53.15243902439025 |
| NaCl0.9MSG0 | 69.23170731707317 |
| NaCl0.9MSG0.3 | 67.4329268292683 |
### Chart
| Category | |
|---|---|
| NaCl0.3MSG0 | 20.491935483870968 |
| NaCl0.3MSG0.3 | 34.54032258064516 |
| NaCl0.6MSG0 | 50.814516129032256 |
| NaCl0.6MSG0.3 | 50.78225806451613 |
| NaCl0.9MSG0 | 67.28225806451613 |
| NaCl0.9MSG0.3 | 64.25806451612904 |
### Chart
| Category | |
|---|---|
| NaCl0.3MSG0 | 28.701923076923077 |
| NaCl0.3MSG0.3 | 34.58653846153846 |
| NaCl0.6MSG0 | 53.88461538461539 |
| NaCl0.6MSG0.3 | 53.69230769230769 |
| NaCl0.9MSG0 | 74.89423076923077 |
| NaCl0.9MSG0.3 | 69.11538461538461 |c
d
c
d
d
d
c
c
b
b
c
c
b
a
Saltiness (VAS mm)
Saltiness (VAS mm)
Saltiness (VAS mm)
b
a
a
a
NaCl
0.3
0.3
0.6
0.6
0.9
0.9
NaCl
0.3
0.3
0.6
0.6
0.9
0.9
NaCl
0.3
0.3
0.6
0.6
0.9
0.9
MSG
-
+
-
+
-
+
MSG
-
+
-
+
-
+
MSG
-
+
-
+
-
+
Fukuoka (N=40)
Nagasaki (N=35)
Okinawa (N=35)
### Chart
| Category | |
|---|---|
| NaCl0.3MSG0 | 25.325 |
| NaCl0.3MSG0.3 | 39.58125 |
| NaCl0.6MSG0 | 55.79375 |
| NaCl0.6MSG0.3 | 58.39375 |
| NaCl0.9MSG0 | 74.81875 |
| NaCl0.9MSG0.3 | 75.40625 |
### Chart
| Category | |
|---|---|
| NaCl0.3MSG0 | 23.585714285714285 |
| NaCl0.3MSG0.3 | 32.27857142857143 |
| NaCl0.6MSG0 | 52.17857142857143 |
| NaCl0.6MSG0.3 | 53.535714285714285 |
| NaCl0.9MSG0 | 71.41428571428571 |
| NaCl0.9MSG0.3 | 68.27857142857142 |
### Chart
| Category | |
|---|---|
| NaCl0.3MSG0 | 23.264285714285716 |
| NaCl0.3MSG0.3 | 35.75714285714286 |
| NaCl0.6MSG0 | 56.92142857142857 |
| NaCl0.6MSG0.3 | 54.714285714285715 |
| NaCl0.9MSG0 | 72.5 |
| NaCl0.9MSG0.3 | 68.79285714285714 |d
d
c
d
c
d
c
b
c
c
c
b
b
Saltiness (VAS mm)
Saltiness (VAS mm)
b
Saltiness (VAS mm)
a
a
a
a
NaCl
0.3
0.3
0.6
0.6
0.9
0.9
NaCl
0.3
0.3
0.6
0.6
0.9
0.9
NaCl
0.3
0.3
0.6
0.6
0.9
0.9
MSG
-
+
-
+
-
+
MSG
-
+
-
+
-
+
MSG
-
+
-
+
-
+
P < 0.0001*
P < 0.0001*
P < 0.0001*
P < 0.0001*
P < 0.0001*
P < 0.0001*
P < 0.0001*
P < 0.0001*
P < 0.0001*
P < 0.0001*
P < 0.0001*
P < 0.0001*
Supp. Fig. 2–A Mean and SE of saltiness VAS rating among the health seminar participants
The ratings of saltiness of the six samples among 327 participants and at each of the eleven health seminars. The significant differences between each rating score are indicated by alphabetic superscripts. A column is significantly different from others that do not have the same alphabetic superscript according to Tukey’s test (P<0.05). *: P for repeated-measures ANOVA.

## Slide 4
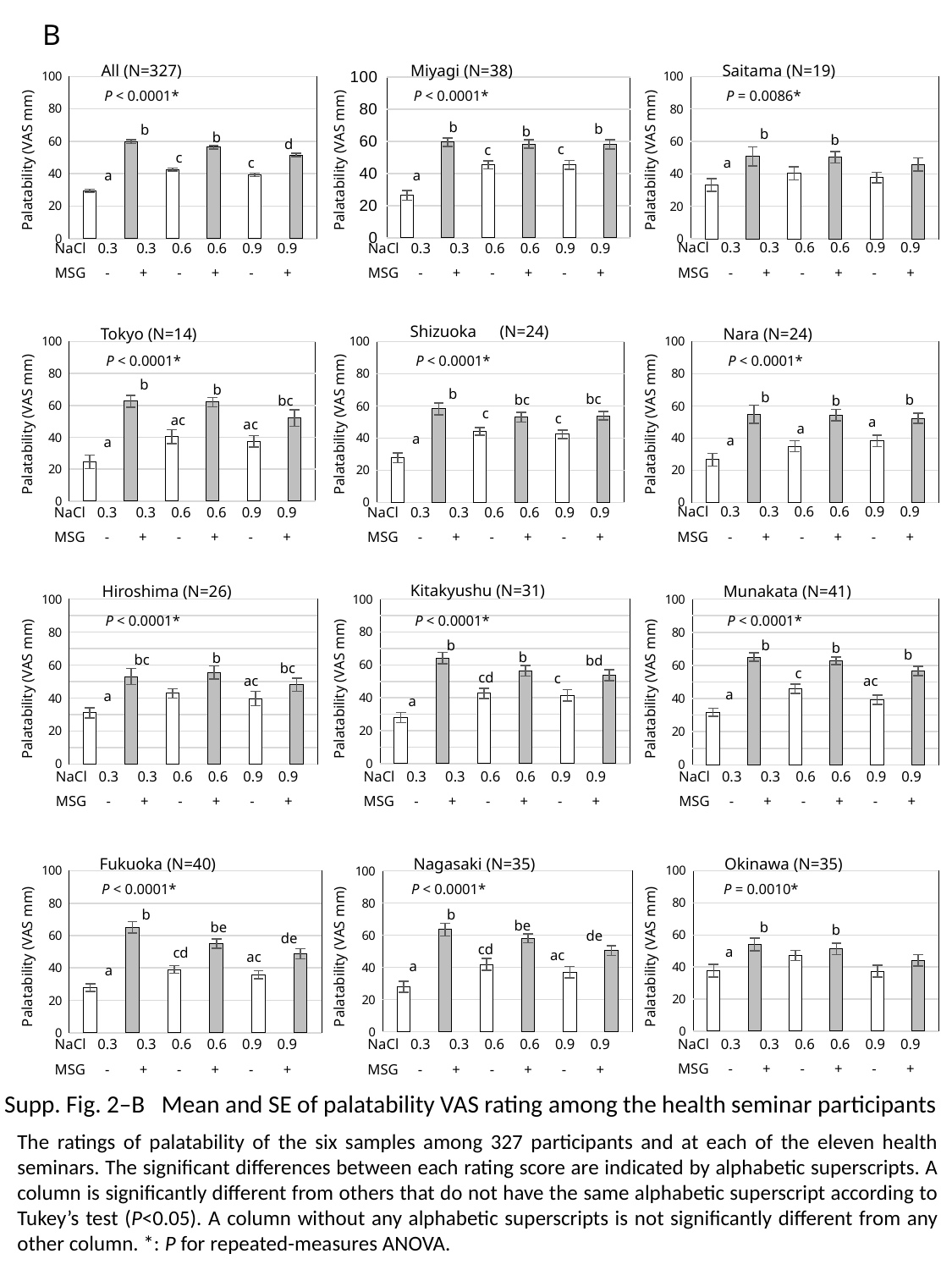

B
All (N=327)
Miyagi (N=38)
Saitama (N=19)
### Chart
| Category | |
|---|---|
| NaCl0.3MSG0 | 26.348684210526315 |
| NaCl0.3MSG0.3 | 59.46052631578947 |
| NaCl0.6MSG0 | 45.32236842105263 |
| NaCl0.6MSG0.3 | 58.38815789473684 |
| NaCl0.9MSG0 | 45.31578947368421 |
| NaCl0.9MSG0.3 | 58.05921052631579 |
### Chart
| Category | |
|---|---|
| NaCl0.3MSG0 | 29.530733944954125 |
| NaCl0.3MSG0.3 | 59.797400611620795 |
| NaCl0.6MSG0 | 42.620795107033636 |
| NaCl0.6MSG0.3 | 56.28593272171254 |
| NaCl0.9MSG0 | 39.305810397553515 |
| NaCl0.9MSG0.3 | 51.50152905198777 |
### Chart
| Category | |
|---|---|
| NaCl0.3MSG0 | 33.10526315789474 |
| NaCl0.3MSG0.3 | 50.69736842105263 |
| NaCl0.6MSG0 | 40.276315789473685 |
| NaCl0.6MSG0.3 | 50.19736842105263 |
| NaCl0.9MSG0 | 37.63157894736842 |
| NaCl0.9MSG0.3 | 45.776315789473685 |b
b
b
b
b
b
b
d
c
c
c
Palatability (VAS mm)
Palatability (VAS mm)
Palatability (VAS mm)
c
a
a
a
NaCl
0.3
0.3
0.6
0.6
0.9
0.9
NaCl
0.3
0.3
0.6
0.6
0.9
0.9
NaCl
0.3
0.3
0.6
0.6
0.9
0.9
MSG
-
+
-
+
-
+
MSG
-
+
-
+
-
+
MSG
-
+
-
+
-
+
Shizuoka　(N=24)
Tokyo (N=14)
Nara (N=24)
### Chart
| Category | |
|---|---|
| NaCl0.3MSG0 | 24.589285714285715 |
| NaCl0.3MSG0.3 | 62.535714285714285 |
| NaCl0.6MSG0 | 40.267857142857146 |
| NaCl0.6MSG0.3 | 62.05357142857143 |
| NaCl0.9MSG0 | 37.375 |
| NaCl0.9MSG0.3 | 52.05357142857143 |
### Chart
| Category | |
|---|---|
| NaCl0.3MSG0 | 27.666666666666668 |
| NaCl0.3MSG0.3 | 58.125 |
| NaCl0.6MSG0 | 44.125 |
| NaCl0.6MSG0.3 | 52.885416666666664 |
| NaCl0.9MSG0 | 42.3125 |
| NaCl0.9MSG0.3 | 53.84375 |
### Chart
| Category | |
|---|---|
| NaCl0.3MSG0 | 26.5 |
| NaCl0.3MSG0.3 | 54.78125 |
| NaCl0.6MSG0 | 34.84375 |
| NaCl0.6MSG0.3 | 54.208333333333336 |
| NaCl0.9MSG0 | 38.104166666666664 |
| NaCl0.9MSG0.3 | 52.270833333333336 |b
b
b
b
bc
b
bc
bc
b
c
c
ac
a
ac
Palatability (VAS mm)
Palatability (VAS mm)
Palatability (VAS mm)
a
a
a
a
NaCl
0.3
0.3
0.6
0.6
0.9
0.9
NaCl
0.3
0.3
0.6
0.6
0.9
0.9
NaCl
0.3
0.3
0.6
0.6
0.9
0.9
MSG
-
+
-
+
-
+
MSG
-
+
-
+
-
+
MSG
-
+
-
+
-
+
Kitakyushu (N=31)
Hiroshima (N=26)
Munakata (N=41)
### Chart
| Category | |
|---|---|
| NaCl0.3MSG0 | 31.01923076923077 |
| NaCl0.3MSG0.3 | 53.02884615384615 |
| NaCl0.6MSG0 | 42.77884615384615 |
| NaCl0.6MSG0.3 | 55.52884615384615 |
| NaCl0.9MSG0 | 39.69230769230769 |
| NaCl0.9MSG0.3 | 48.11538461538461 |
### Chart
| Category | |
|---|---|
| NaCl0.3MSG0 | 27.862903225806452 |
| NaCl0.3MSG0.3 | 64.08064516129032 |
| NaCl0.6MSG0 | 42.685483870967744 |
| NaCl0.6MSG0.3 | 56.32258064516129 |
| NaCl0.9MSG0 | 41.443548387096776 |
| NaCl0.9MSG0.3 | 53.66935483870968 |
### Chart
| Category | |
|---|---|
| NaCl0.3MSG0 | 31.682926829268293 |
| NaCl0.3MSG0.3 | 65.02439024390245 |
| NaCl0.6MSG0 | 45.84146341463415 |
| NaCl0.6MSG0.3 | 62.80487804878049 |
| NaCl0.9MSG0 | 39.329268292682926 |
| NaCl0.9MSG0.3 | 56.5609756097561 |b
b
b
b
b
b
bc
bd
bc
c
cd
c
ac
ac
Palatability (VAS mm)
Palatability (VAS mm)
Palatability (VAS mm)
a
a
a
NaCl
0.3
0.3
0.6
0.6
0.9
0.9
NaCl
0.3
0.3
0.6
0.6
0.9
0.9
NaCl
0.3
0.3
0.6
0.6
0.9
0.9
MSG
-
+
-
+
-
+
MSG
-
+
-
+
-
+
MSG
-
+
-
+
-
+
Fukuoka (N=40)
Nagasaki (N=35)
Okinawa (N=35)
### Chart
| Category | |
|---|---|
| NaCl0.3MSG0 | 27.75 |
| NaCl0.3MSG0.3 | 64.99375 |
| NaCl0.6MSG0 | 39.05 |
| NaCl0.6MSG0.3 | 55.125 |
| NaCl0.9MSG0 | 35.875 |
| NaCl0.9MSG0.3 | 48.6875 |
### Chart
| Category | |
|---|---|
| NaCl0.3MSG0 | 28.035714285714285 |
| NaCl0.3MSG0.3 | 63.50714285714286 |
| NaCl0.6MSG0 | 41.77142857142857 |
| NaCl0.6MSG0.3 | 58.01428571428571 |
| NaCl0.9MSG0 | 36.91428571428571 |
| NaCl0.9MSG0.3 | 50.41428571428571 |
### Chart
| Category | |
|---|---|
| NaCl0.3MSG0 | 37.75857142857143 |
| NaCl0.3MSG0.3 | 54.05714285714286 |
| NaCl0.6MSG0 | 47.18571428571428 |
| NaCl0.6MSG0.3 | 51.25 |
| NaCl0.9MSG0 | 37.32857142857143 |
| NaCl0.9MSG0.3 | 44.107142857142854 |b
b
be
b
be
b
de
de
cd
a
cd
ac
ac
Palatability (VAS mm)
Palatability (VAS mm)
Palatability (VAS mm)
a
a
NaCl
0.3
0.3
0.6
0.6
0.9
0.9
NaCl
0.3
0.3
0.6
0.6
0.9
0.9
NaCl
0.3
0.3
0.6
0.6
0.9
0.9
MSG
-
+
-
+
-
+
MSG
-
+
-
+
-
+
MSG
-
+
-
+
-
+
P < 0.0001*
P < 0.0001*
P = 0.0086*
P < 0.0001*
P < 0.0001*
P < 0.0001*
P < 0.0001*
P < 0.0001*
P < 0.0001*
P < 0.0001*
P < 0.0001*
P = 0.0010*
Supp. Fig. 2–B Mean and SE of palatability VAS rating among the health seminar participants
The ratings of palatability of the six samples among 327 participants and at each of the eleven health seminars. The significant differences between each rating score are indicated by alphabetic superscripts. A column is significantly different from others that do not have the same alphabetic superscript according to Tukey’s test (P<0.05). A column without any alphabetic superscripts is not significantly different from any other column. *: P for repeated-measures ANOVA.
.
